# Supplementary material for: Clarifying the structure of serious head and spine injury in youth Rugby Union players
Source: PLoS One. 2020 Jul 15;15(7):e0235035. doi: 10.1371/journal.pone.0235035 (PMC7363091; doi:10.1371/journal.pone.0235035)
Supplement: Attachment — Submitted filename: response_to_ reviewer_ks20200325.docx [file pone.0235035.s001.docx]

In response to the all reviewer's thankful comments, I have significantly modified, Tables, introduction, especially in the methods of network analysis, results (table amendments), discussions, and conclusions.

20200321

**Reviewer 1**

The title is not quite understandable.

＞＞＞We changed the tittle.

the introduction lacks on focusing the key constructs and does not tackle adequately why using network theory and specifically betweenness centrality. It is too much generalised and focusing on things that does not have relevance for the topic. The subsection Network Analysis title is repeated in the results, and in the first part it does not explain why using bipartite networks, betweenness centrality (after referring clustering and random walks). This subsection is quite confusing on the relevance of the methods and metrics chosen. In the section Statistical Analysis, the results does not seem to follow a sequence, repeating the subsection of Network Analysis.

　　＞＞＞In Introduction and methods, we explained rugby tackle and network analysis approaches、bipartite networks, betweenness centrality　precisely.

In the Discussion section, there is some information text should be on the method section (e.g. lines 283-285, 300-305, 306).

>>>We sifted those to method section.

The conclusion section does not reflect the importance of the study, regarding suggestions and future changes of the rules of the game. Its also confusing on the structure.

>>>In discussion and conclusion section, we added our study suggestion that we should consider introducing rules that prohibit head-on collisions in youth rugby.

**Reviewer 2**

1 - Although the quality of written language is reasonable, it could benefit largely from a revision from a native speaker. The text is understandable, but sometimes difficult to follow. There are also many repetitions and some confusing sentences.

>>>Our paper was checked by native English speaker.

2 - The authors should advance proposals - even if speculative - to circumvent what is occurring with the younger and/or less experienced players. What can be done to improve the situation and make them less exposed to such injuries? Could potential rules changes help? Again, even if speculative, it could provide working ideas for concerning parties, as well as generate interesting and novel prospective studies.

>>>We added text suggest to prevent head collision risk on young rugby players in nearl future.

**Reviewer 3**

Appreciate many meaningful comments.

- The abstract is very poor. The authors do not refer the methods and/or statistical procedures used for analysing the data, and do not provide any results;

>>>> We changed Abstract more detailed explanations.

- The study has no previous established hypotheses;

>>>In Introduction and methods section, we mentioned our study hypotheses and explained our rugby injuries prevention approach structure by network analysis.

-The Introduction section is almost (not to say entirely) directed to the properties of the tool/methods (network analysis) and its applications. It is not clear the real relevance of this research if the authors only focus on the tool. In the methods section, the authors need to provide more details regarding the underlying procedures of a bipartite graph (definition, methods/algorithms, etc). Which social network software, or other tool (please mention the characteristics of the software or tool) was used to create the bipartite network? How was centrality and edge-betweenness centrality calculated? This is important, because, depending on the software (if such a software was used), each one has its own way to calculate centrality-based indicators;
Moreover, why did the authors chose the edge-betweenness centrality and how it is defined/calculated? There is missing information regarding the modularity Q (how the cluster is divided, cut-off points? Software that was used to calculate (Matlab?), etc);

>>> We described why we chose bipartite graph, edge-betweenness centrality, R igraph program, precisely.

The three first paragraphs of the Discussion section have a strong message regarding the mismatch situations that typically occur in sports performance, and it would be highly relevant to re-write the introduction based, not only on methods,

>>> We explained those in the introduction too.

- The Results section is dense. There are lots of tables which can hinder or even distract readers attention. Is there any way that the authors can combine tables in order to reduce their number? For example, the tables depicting the number of injuries, causes, etc, can be amalgamated in only one table;

- The authors do not mention the aim of their study in the first paragraph of the Discussion section, before starting to discuss their results. Moreover, by defining previous hypotheses there is a need to report if they were or not confirmed. Importantly, the authors need to include more references to support their findings;

>>>>Result section, we change our tables and Discussion section, we referred some other studies.

- The Discussion section can be enriched by providing a more in-depth examination and discussion concerning the mismatch in cross-age and cross-body size of players. Undoubtedly, these encompass two potent messages for the scientific community that can impact in the way that sports organizations and sports federations organize competitions;

>>>We mentioned our hypotheses and aim and our conclusion and suggestion was described as we should consider introducing rules that prohibit head-on collisions in youth rugby.

- In the conclusion section please report what type of serious injuries do younger and inexperienced players suffer.

>>> In Conclusion section, we added what type of serious injuries do younger and inexperienced players suffer.

We send deep appreciation for all three reviewers.

with very best regards,
