# Supplementary material for: Clarifying the structure of serious head and spine injury in youth Rugby Union players
Source: PLoS One. 2020 Jul 15;15(7):e0235035. doi: 10.1371/journal.pone.0235035 (PMC7363091; doi:10.1371/journal.pone.0235035)
Supplement: Attachment — Submitted filename: PLOS ONE_review.docx [file pone.0235035.s002.docx]

**PLOS ONE**

**General appreciation**

The manuscript entitled “Clarifying the structure of serious head and spine injury in youth Rugby Union players.”, intends to clarify the cause of rugby head and spinal cord injuries through a network centrality analysis of 14-year (2004-2018) longitudinal data in Japan. The authors implemented a network bipartite graph and subgroup (cluster) analysis to examine injured players' experience and the caused of injury. The manuscript is in general well written and provides some interesting points with respect to the aim of the authors. The topic of the manuscript is relevant and adequate for the journal. Regardless, there are some issues that still need to be address before the publication can be considered.

**The manuscript has the following strong points:**

- The manuscript uses longitudinal data and provides an interesting view of how network analysis can be applied to examine rugby head and spinal cord injuries, which can be further extended to other team sports;

**The manuscript has the following weak points:**

- In the Introduction section, the authors perform a brief literature review regarding the application of the network analysis in different disciplines (economics, physics, social, etc). However, the authors over-emphasise technical aspects of the network analysis and there is little information concerning its applications on team sports like rugby. What does previous research tells us about the application of the network analysis in rugby? Is there any study that have tried to use social networks to examine injuries? What are the common limitations found in previous studies, beyond an over-emphasis on transversal studies than longitudinal?

- In line 70 of the Introduction section the authors report the importance of network dynamics. What is the difference between a static network and a dynamic network? What about this study? It focuses on static networks or dynamic networks? How does network analysis evaluate dynamic networks?

- The authors report that networks can be used to identify global properties of the network (e.g., structures that emerge more often) as well as local properties (identification of individuals who have a key role in the network). But, what about in this study? Typically, the network analysis conceives the individuals as the network nodes and their relationships are commonly evaluate, for example, through ball-passing actions. In this study, what are the nodes of the network and what type of connections are established among nodes. Although it is implicit throughout the manuscript, the authors need to clarify this. Not all readers are familiar with this type of analysis.

- In the Methods section, there are lots of tables and much information. Is there any way of collapsing information from tables in only two or three tables maximum?

- What are the nodes of the network and what are the links? Is this a static or dynamic network? Why using the edge-betweenness centrality? Is there any limitations of this metric? Please clarify this.

- How were the causes of injuries categorized?

- Please report the aim of the study in the first paragraph of the Discussion section.

- The study hypothesis is very general. Are the authors trying to analyse if the occurrence of serious injuries is related with player experience? Please clarify and make it more specific.

- The authors refer: “Quality of experience”. Is it quality of experience or quantity of experience (i.e. accumulated experience according to the number of years playing rugby)?

- In the Discussion section the authors refer that the data could contribute in preventing serious injuries in rugby players. How? Is the authors aim to signalise the attention of governments and elite rugby organizations in terms of sports policies that can prevent these injuries?

Given the aforementioned, it is my recommendation that the manuscript should not be considered for publication in the current form. However, I am looking forward to see another draft of the paper.
