# Supplementary material for: Clarifying the structure of serious head and spine injury in youth Rugby Union players
Source: PLoS One. 2020 Jul 15;15(7):e0235035. doi: 10.1371/journal.pone.0235035 (PMC7363091; doi:10.1371/journal.pone.0235035)
Supplement: Attachment — Submitted filename: Answer_for_reviewer20200531.docx [file pone.0235035.s003.docx]

Answre to the reviewer

Q. - In the Introduction section, the authors perform a brief literature review regarding the application of the network analysis in different disciplines (economics, physics, social, etc). However, the authors over-emphasise technical aspects of the network analysis and there is little information concerning its applications on team sports like rugby. **What does previous research tells us about the application of the network analysis in rugby?**

We added next study. Sorry it was related soccer (we could not find Rugby analysis studies)

A. It was considered that there was a central player called a hub in the passing behavior research of a soccer game, and there was a power law there. Furthermore, the hub dynamically switched throughout the game. The difference between dynamic networks and static networks is that the former focuses on the variable and diverse play structures occurring in sports games.[18] (page.5 line.72 )

**Q. Is there any study that have tried to use social networks to examine injuries?**

We added next study.

A. The factors that play a central role in various physiological parameters during exercise-induced fatigue were clarified by network analysis, and its application to risk management was discussed [13]. (page.4	Line.68 )

**Q. What are the common limitations found in previous studies, beyond an over-emphasis on transversal studies than longitudinal?**

A. The reason why there are few longitudinal studies would be that the social rules for ethical use of medical diagnosis and for protecting personal information were not sufficiently established. (page.3	line.44	)

Q. - In line 70 of the Introduction section the authors report the importance of network dynamics. What is the **difference between a static network and a dynamic network?**

A. The difference between dynamic networks and static networks is that the former focuses on the variable and diverse play structures occurring in sports games [18]. (p.5	L:74 )

**What about this study? It focuses on static networks or dynamic networks? How does network analysis evaluate dynamic networks?** - The authors report that networks can be used to identify global **properties** of the network (e.g., structures that emerge more often) as well as local properties (identification of individuals who have a key role in the network). But, what about in this study? Typically, the network analysis conceives the individuals as the network nodes and their relationships are commonly evaluate, for example, through ball-passing actions. In this study, what are the **nodes** of the network and what type of connections are established among nodes. Although it is implicit throughout the manuscript, the authors need to clarify this. Not all readers are familiar with this type of analysis.

**Nodes** of network in this study are player groups (age and years of experience), types of injuries, and play styles that cause injuries (tackle, tackled, scrum, etc.). It clarifies the continuity of what kind of players’ experience years, what kind of play, and what part of body collision caused serious injury. These are the **dynamic** networks which **focus on the diverse play structures occurring throughout rugby game**. Although this study would clarify a kind of **local properties** of networks, it might be considered as global properties by promoting international joint study in the future. (p7, L:119)

- In the Methods section, there are **lots of tables** and much information. Is there any way of collapsing information from tables in only two or three tables maximum?

We change from 9 tables to 4 tables. (p10: table 1,2, p12:Table3, p14:Table4)

- What are the **nodes** of the network and what are the **links**? Is this a static or dynamic network?

Same adobe

**Nodes** of network in this study are player groups (age and years of experience), types of injuries, and play styles that cause injuries (tackle, tackled, scrum, etc.). It clarifies the **continuity** of what kind of players’ experience years, what kind of play, and what part of body collision caused serious injury. These are the **dynamic** networks which **focus on the diverse play structures occurring throughout rugby game**. Although this study would clarify a kind of **local properties** of networks, it might be considered as global properties by promoting international joint study in the future. (p7. L.119)

W**hy using the edge-betweenness centrality? Is there any limitations of this metric? Please clarify this.**

A. Edge-betweenness centrality represents the degree of being located in the shortest path bridge between nodes. It was adopted because it would indicate a close relationship among players, injuries, and the causes. The limitation of this metric is that its approximate value may become unstable in case of a large-scale network graph [27, 28]. (p8 L:136)

- How were the causes of injuries categorized?

A.These classification processes were performed by the safety management committee of the Japanese Rugby Football Union. This committee works closely with the medical committee, coaching committee, referee committee, and technical committee in Japanese Rugby Union, cooperates in classification of the cause of injury based on the detailed play situation reported by the team manager and doctor. (p.7 l.112 )

- Please report the aim of the study in the first paragraph of the Discussion section.- The study hypothesis is very general. Are the authors trying to analyse if the occurrence of serious injuries is related with player experience? Please clarify and make it more specific.

We change the sentense under bellow.

A. The study hypothesis was to clarify the fact that the causes of serious rugby injuries such as head injury and spinal cord injury are due to the years of player experience. That knowledge could help to propose practical rules for injury prevention. (p.18	L:300 )

- The authors refer: “Quality of experience”. Is it quality of experience or quantity of experience (i.e. accumulated experience according to the number of years playing rugby)?

We change the sentense

The study hypothesis was to clarify the fact that the causes of serious rugby injuries such as head injury and spinal cord injury are due to **the years of player experience**. (P18, L:301)

- In the Discussion section the authors refer that the data could contribute in preventing serious injuries in rugby players. How? Is the authors aim to signalise the attention of governments and elite rugby organizations in terms of sports policies that can prevent these injuries?

**We added the sentense under bellow.**

Our study suggests that we should consider introducing rules that prohibit head-on collisions in youth inexperienced rugby players. Such specific rules could be developed in collaboration with medical, refereeing, technical and coaching experts. The injury prevention guidelines would also signalize the attention of governments (Japan Sports Agency, Japan Sports Council) and elite rugby organizations (World Rugby) in terms of sports policies that can prevent these serious injuries. (p. 19 L:313	)
